# Retinal Vessel Density Loss Without Structural Thinning in Early Multiple Sclerosis: An Optical Coherence Tomography Angiography Study

**DOI:** 10.1007/s44448-026-00006-w

**Published:** 2026-11-03

**Authors:** Elizabeth Silbermann, Ou Tan, Jie Wang, Andrea Hildebrand, Lindsey Wooliscroft, Emma Young, Yali Jia, Po-Han Yeh, Rebecca Spain, Dennis Bourdette, David Huang

**Affiliations:** 1 Department of Neurology, Oregon Health & Science University, Portland, OR USA; 2 VA Portland Health Care System, Portland, OR USA; 3 Casey Eye Institute, Oregon Health & Science University, Portland, OR USA; 4 Biostatistics & Design Program, Oregon Health & Science University, Portland, OR USA; 5 Division of Informatics, Clinical Epidemiology, and Translational Data Science, Oregon Health & Science University, Portland, OR USA; 6 Department of Ophthalmology, New Taipei Municipal Tucheng Hospital, New Taipei City, Taiwan

**Keywords:** Multiple sclerosis, Optical coherence tomography angiography, Retinal microvasculature, Optic neuritis, Neurodegeneration, Vascular biomarkers

## Abstract

Retinal microvascular and structural changes both occur in multiple sclerosis (MS), but their relationship in early disease remains unclear. We conducted a prospective observational study of 15 participants with early relapsing-remitting MS (RRMS; mean disease duration 1.1 years) and 30 age- and sex-matched healthy controls (HC), using optical coherence tomography angiography (OCTA) to compare retinal vessel density and structural thickness. MS eyes were stratified by optic neuritis (ON) history. MS eyes without ON history (MS-NON) showed a significant (all standardized β < −1, *p* < 0.001) reduction in vessel density as compared to HC in macular superficial vascular complex (SVC) and ganglion cell layer plexus (GCLP), as well as peripapillary nerve fiber layer plexus (NFLP), despite relatively preserved macular ganglion cell inner plexiform layer (GCIPL β = −0.26) and ganglion cell complex thickness (GCC β = −0.17; both non-significant). In contrast, MS eyes with ON history (MS-ON) showed tightly coupled structural thinning and reduced vessel density. Structure-vascular correlations in MS-NON eyes were weak for superficial compartments (GCLP, SVC) but strong for NFLP. These findings indicate that microvascular dysfunction can occur in the absence of detectable structural loss in early MS, and raise the possibility that, in eyes without optic neuritis, vascular changes may precede detectable neuronal loss. OCTA-derived vascular biomarkers may complement structural OCT in early MS monitoring.

## Introduction

Multiple sclerosis (MS) is a chronic inflammatory and neurodegenerative disease of the central nervous system affecting approximately 1 million people in the United States [1]. Vascular dysfunction contributes to MS pathology through several mechanisms. Blood-brain barrier disruption allows inflammatory cells to infiltrate the central nervous system, cerebrovascular dysregulation leads to reduced perfusion and metabolic support, and oxidative stress leads to neuronal death [2, 3]. Retinal vascular imaging with optical coherence tomography angiography (OCTA) allows non-invasive assessment of microvascular density, which may reflect downstream sequelae of these broader pathological processes.

The retina provides a unique window into central nervous system pathology through non-invasive imaging. Optical coherence tomography (OCT) can detect atrophy of the retinal nerve fiber layer (RNFL) and ganglion cell-inner plexiform layer (GCIPL) in both relapsing-remitting [4] and progressive MS [5, 6], which correlates with brain atrophy [7] and predicts disability independent of past optic neuritis (ON) [8]. OCTA extends this by quantifying retinal microvasculature [9, 10]. Meta-analyses confirm reduced vessel density in MS [11, 12] but whether these vascular changes cause or result from neuronal loss is unknown.

Emerging literature suggests that microvascular changes in MS can be detected even when structural changes in the RNFL and GCIPL are subtle or absent. Using deep learning analyses, Wicklein et al. identified rarefaction (reduction in vessel density) of small-sized vessels (diameter < 10 μm) in MS eyes independent of ON history, which was associated with greater clinical disability as well as longer disease duration [13]. However, because of the cross-sectional nature of the study, the temporal relationship between vessel density and structural changes remains unclear.

To determine whether retinal vessel density is reduced in the absence of macular ganglion cell thinning in early MS, we conducted a prospective single-arm, observational study of early RRMS with cross-sectional comparison to age- and sex-matched healthy controls (HC) from repository data. Participants were stratified by ON history to compare retinal structural and microvascular measures. Secondary aims were to evaluate correlations between retinal vascular and structural measures and clinical disability, and to evaluate longitudinal changes over 6-month follow-up in the RRMS cohort. We hypothesized that retinal microvascular differences would be detectable in MS eyes without ON history as compared to HC even when structural thickness is relatively preserved, which would suggest that microvascular dysfunction can occur independently of neuronal loss in early MS.

## Methods

### Participants

This study was approved by the Institutional Review Board at Oregon Health & Science University (OHSU); written informed consent was obtained from all participants. Enrollment was from January 13, 2021 to November 5, 2021, and the last participant exited August 21, 2022.

Fifteen participants with RRMS were recruited from OHSU MS Clinic and enrolled in a 6-month, prospective, observational, single-arm study evaluating OCTA and visual function. Inclusion criteria were: age 18–70 years with diagnosis of clinically isolated syndrome or MS by 2017 McDonald Criteria within 5 years of study enrollment. Participants were evaluated at four time points: baseline, months 1, 3, and 6. Baseline and longitudinal OCT/OCTA data from 30 age- and sex-matched HC were obtained from a clinical research repository as part of the “Functional and Structural Optical Coherence Tomography for Glaucoma” Study [14] at OHSU and included in the primary analysis.

Exclusion criteria for both MS and HC included: significant ophthalmological pathology (glaucoma, diabetic retinopathy, hypertensive retinal disease, age-related macular degeneration, uveitis, amblyopia), spherical equivalent refractive error > ± 3 diopters or astigmatism > 2 diopters, previous intraocular surgery except cataract extraction, or major medical/neurological comorbidity other than MS.

Prior optic neuritis was ascertained by patient-reported clinical history at enrollment and, where records were available, verified by review of the medical record; no confirmatory magnetic resonance imaging or visual evoked potential data were available. ON history was reconfirmed by clinical history at each follow-up visit. Recent optic neuritis was not an exclusion criterion.

### Imaging Protocol

Retinal imaging was performed using a 120-kHz spectral-domain OCT system (Optovue Solix; Visionix, Fremont, CA, USA) with integrated OCTA (split-spectrum amplitude-decorrelation angiography algorithm [15]) following pupil dilation with tropicamide 1% and phenylephrine 2.5%. Imaging was performed in a standardized room setting. Scans included a 6 × 6 mm OCTA scan centered on the optic disc and a 6.4 mm×6.4 mm OCTA scan centered on the fovea. Retinal layers including the inner limiting membrane, posterior boundary of the nerve fiber layer, and posterior boundary of the inner plexiform layer, were segmented using the algorithm embedded in the Solix ReVue software (V1.0.0.342; Visionix/Optovue), followed by manual boundary correction using customized grading software (COOL-ART) equipped with an intelligent manual correction module [16]. En face angiograms were generated using maximum value projection within the specified slabs [17]. The NFLP extended from the inner limiting membrane to the posterior boundary of the nerve fiber layer and was measured in the peripapillary scan [14]. In the macular scan, the GCLP extended from the posterior boundary of the nerve fiber layer to 75% of the depth of the ganglion cell-inner plexiform layer, the watershed between the GCLP and the intermediate capillary plexus, and the SVC extended from the inner limiting membrane to the same depth, combining the NFLP and GCLP [18]. Finally, overall OCT and OCTA parameters were generated from 4.5 × 4.5 mm² peripapillary regions (centered on the optic nerve head) and 6 × 6 mm² macular regions (centered on the fovea).

Measurement domains included: (1) Structural thickness measures (OCT): Ganglion Cell Complex (GCC), GCIPL, and RNFL thickness; and (2) Vessel density (VD) measures (OCTA): Ganglion Cell Layer Plexus (GCLP), Superficial Vascular Complex (SVC), and Nerve Fiber Layer Plexus (NFLP). GCC, GCIPL, GCLP and SVC are derived from macular scans; RNFL and NFLP are derived from peripapillary scans centered on the optic nerve head. Participants with early MS were selected to minimize retinal atrophy seen in established disease [19]. VD is reported as an automated software calculation of the percentage of perfused pixels within each vascular layer using Solix software (V1.0.0.342; Visionix). All OCT and OCTA values reported here are whole-region averages over these analysis areas; no sector-level or subfield analyses were performed. The analysis areas are nested within the acquired scans, with peripapillary parameters derived from the 4.5 × 4.5 mm² region of the 6 × 6 mm optic disc scan and macular parameters from the 6 × 6 mm² region of the 6.4 × 6.4 mm foveal scan.

Quality control procedures followed APOSTEL (Advised Protocol for OCT Studies) [20] and OSCAR-MP consensus guidelines [21]. Participants received at least two replicate scans per visit. Images were reviewed for motion artifact, projection artifact, signal loss, and segmentation errors by a single observer (Grader PY). Only scans with a signal strength index (SSI) > 50 and a shadow percentage < 10% were included in the analysis. Twelve scans (2.2%) were excluded due to low quality.

### Clinical Assessments

MS participants completed clinical testing at baseline including timed 25-Foot Walk Test (T25FW), written Symbol Digit Modalities Test (SDMT) [22, 23], and Nine-Hole Peg Test (9HPT) [24]. The MS Functional Composite (MSFC) [25–27] was calculated from these measures using published MS normative data for each component. Measures of visual function included Early Treatment Diabetic Retinopathy Study [28] (ETDRS) best-corrected visual acuity (BCVA) and Sloan low-contrast letter acuity (LCLA) at 5%, 2.5%, and 1.25%. A Winsorized estimator was used for participants unable to complete vision testing due to poor acuity, assigning them a score of 1.1 (equivalent to 20/250 vision). Patient-reported disability was evaluated using Patient-Determined Disability Steps [29] (PDDS) and National Eye Institute Visual Function Questionnaire-25 (VFQ-25) as well as the Neuro-Ophthalmic Supplement [30, 31].

### Statistical Analysis

Thirty age- and sex-matched controls were selected from the total sample of 76 individuals with complete OCT and OCTA data contained in the research repository. HC were matched 2:1 to MS participants using a multivariate matching algorithm prioritizing age (± 4 years), with additional optimization for sex distribution by nearest neighbor matching on propensity score using the R package MatchIt [32]. Given the demographic homogeneity of the MS cohort (86.7% female, 93.3% white) compared to the pool of healthy controls (53.9% female and 63.2% white), we prioritized the need for tight age matching over improved sex and race representation when selecting the matched sample.

To correct for axial length, SSI, and age, multivariable regression models were constructed for each OCT/OCTA parameter to identify confounding by these covariates (*p* < 0.05). The resulting coefficients of confounding covariates were used to calculate a mean-centered correction factor for each OCT and OCTA measure, which was applied before conducting any analyses.

For all eye-level analyses, each eye was classified as HC, MS eye with prior ON (MS-ON) and MS eye with no prior ON (MS-NON). For all person-level analyses, each person is classified as HC, MS-ON (if any history of ON in either eye), or MS-NON (if no history of ON in either eye).

To analyze cross-sectional differences between matched controls and MS eyes with and without history of ON, we constructed mixed effects linear regression models for each OCT and OCTA measure. Each model took eye as the unit of assessment and used all available scans per participant at baseline, up to 4 replicate scans. Models included random effects for participant and eye within participant and fixed effects for group (HC, MS-ON, MS-NON) and sex. Model coefficients were standardized to enable comparison of effect sizes between the various OCT and OCTA measures. Each outcome measure was z-scored prior to modeling, so that a standardized coefficient (β) of 1.0 corresponds to a one-standard-deviation difference in that measure; group differences are therefore reported as standardized β coefficients expressed in standard-deviation units. For each comparison we also report the percent difference between adjusted group means relative to the healthy control mean, alongside the standardized β (Table 2b); these adjusted percent differences are not directly reproducible from the unadjusted means in Table 2a.

To assess correlations between OCTA measures, OCT measures, and clinical outcomes in people with MS, we employed an averaging approach to reduce inter-visit measurement variability and improve precision of estimates. For each eye, replicate scans within each visit were first averaged, then values were averaged across all available study visits (up to 4 visits), yielding a single mean value per eye for each OCTA and OCT measure.

To mitigate the impact of inter-eye cluster effects, eye-level correlations were calculated from bootstrapped estimates. The purpose of this approach was to create many views of the data, each containing single eyes per participant, where each view is an unbiased and deliberately incomplete sample of participants and eyes. By averaging over the views, we obtain estimates that by design are unaffected by similarities between eyes within an individual. Bootstrapped samples were created by sampling with replacement from the number of individuals in each of three subgroups (MS-ON eyes, MS-NON eyes, and eyes from the pooled MS cohort), creating three sets of 10,000 samples. From each randomly selected individual within a sample, one eye was randomly selected; in the event of only one eye meeting subgroup criteria (e.g. an individual within the ON subgroup with only one ON eye), the eye meeting criteria was automatically selected. The median and middle 95% of the distribution of the bootstrapped estimates are reported for each correlation under consideration.

Structure-vascular correlations were computed between OCTA VD and OCT structural thickness measures. For eye-level clinical variables (BCVA visual acuity, LCLA), correlations were assessed at the eye level. For person-level variables (VFQ-25, MSFC, PDDS), after averaging as described above, OCTA and OCT measures were further averaged between eyes within each participant, and correlations were assessed at the person level. No bootstrapping was necessary for person-level analysis, as there was no within-person clustering by eye.

Correlations were assessed using Pearson’s correlation coefficient for normally distributed continuous variables and Spearman’s rank correlation coefficient for non-normally distributed or ordinal variables. Associations were considered statistically significant when the 95% confidence interval excluded zero (equivalent to two-tailed *p* < 0.05). Because this study was exploratory and intended to estimate effect sizes and generate hypotheses, we did not correct for multiple comparisons. All comparisons performed are reported in full with effect estimates and 95% confidence intervals (Tables 2, 3, 4 and 5), and no association was selected or emphasized on the basis of statistical significance. As this was a pilot study designed to generate preliminary data for future investigations, we did not perform formal power calculations prior to enrollment.

To assess longitudinal change in MS eyes only, we constructed linear mixed effects regression models for each OCT and OCTA measure. Models included fixed effects for ON status, follow-up time, and ON-by-time interaction, and random effects at the individual and eye levels. Model coefficients were standardized to enable comparison of effect sizes between the various OCT and OCTA measures.

## Results

### Participant Characteristics

Fifteen participants with RRMS and 30 age-matched HC were enrolled (Table 1). Eleven MS participants completed all 4 assessments. Groups were closely matched on age (MS mean 32.6 ± 6.4 years; HC mean 33.7 ± 7.4 years) but differed in sex and racial composition (detailed in Table 1). Among MS participants, mean disease duration was 1.1 (SD 0.9) years, with MS-ON participants having shorter disease duration at 0.9 (SD 1.1) years compared to MS-NON participants at 1.2 (SD 0.9) years. Six participants (40%) had prior ON, affecting nine eyes overall. The median interval between the most recent ON episode and baseline imaging was 4.2 months (range 1.5 to 36.5 months); four participants were imaged within six months of an episode and three within three months. Two participants had two prior ON episodes each; for these participants, the recorded date corresponds to the most recently affected eye. Onset dates were available only to the month for four of the six participants, and the intervals for those participants are therefore approximate. One eye (subject 004, right eye) developed incident optic neuritis between the third and fourth study visits.

**Table 1:** Demographics and Baseline Characteristics

	Healthy Controls	All MS	MS Participant Subgroups by ON Presence	
	(*n* = 30)	(*n* = 15)	MS-NON	MS-ON
			(*n* = 9)	(*n* = 6)
**Age: mean (SD)**	33.7 (7.4)	32.6 (6.4)	33.4 (6.9)	30.2 (6.2)
**Female: n (%)**	17 (56.7)	13 (86.7)	7 (77.8)	6 (100.0)
**Race: n (%)**				
** Asian**	12 (40.0)	0 (0.0)	0 (0.0)	0 (0.0)
** White**	15 (50.0)	14 (93.3)	8 (88.9)	6 (100.0)
** Not reported**	3 (10.0)	1 (6.7)	1 (11.1)	0 (0.0)
**Ethnicity: n (%)**				
** Hispanic**	5 (16.7)	0 (0.0)	0 (0.0)	0 (0.0)
** Non-Hispanic**	16 (53.3)	13 (86.7)	7 (77.8)	6 (100.0)
** Not reported**	9 (30.0)	2 (13.3)	2 (22.2)	0 (0.0)
**Years since diagnosis: mean (SD)**		1.1 (0.9)	1.2 (0.9)	0.9 (1.1)
**DMT at enrollment: n (%)**				
** Anti-CD20 therapy**		7 (46.7)	6 (66.7)	1 (16.7)
** Natalizumab**		2 (13.3)	0 (0.0)	2 (33.3)
** Dimethyl fumarate**		3 (20.0)	1 (11.1)	2 (33.3)
** Siponimod**		1 (6.7)	0 (0.0)	1 (16.7)
** Glatiramer acetate**		1 (6.7)	1 (11.1)	0 (0.0)
** None**		1 (6.7)	1 (11.1)	0 (0.0)
**PDDS: n (%)**				
** 0**		6 (40.0)	4 (44.4)	2 (33.3)
** 1**		4 (26.7)	2 (22.2)	2 (33.3)
** 2**		1 (6.7)	1 (11.1)	0 (0.0)
** 3**		3 (20.0)	1 (11.1)	2 (33.3)
** 4**		1 (6.7)	1 (11.1)	0 (0.0)
**VFQ-25 Composite: mean (SD)**		87.1 (9.0)	89.1 (5.7)	84.1 (12.6)
**VFQ-25 Supplement: mean (SD)**		78.7 (12.1)	83.2 (6.7)	71.9 (15.6)
**T25FW**,** seconds: mean (SD)**		4.6 (1.0)	4.4 (1.1)	4.9 (1.0)
**9HPT**,** seconds**,** dominant hand: mean (SD)**		21.7 (2.1)	21.3 (1.6)	22.3 (2.6)
**SDMT: mean (SD)**		55.5 (7.6)	54.5 (8.1)	57.0 (7.3)
**MSFC: mean (SD)**		0.28 (0.22)	0.27 (0.23)	0.31 (0.21)
			Non-ON Eyes (*n* = 21)	ON Eyes
				(*n* = 9)
**BCVA (LogMAR): median (IQR)**		0.00 (-0.10, 0.10)	0.00 (-0.10, 0.10)	0.00 (0.00, 0.20)
5% LCLA: median (IQR)*		0.30 (0.30, 0.40)	0.30 (0.20, 0.30)	0.40 (0.40, 0.60)
2.5% LCLA: median (IQR)*		0.70 (0.60, 0.80)	0.70 (0.60, 0.70)	0.80 (0.70, 1.10)
1.25% LCLA: median (IQR)*		0.80 (0.70, 1.00)	0.70 (0.70, 0.90)	0.90 (0.80, 1.10)

MS-NON = multiple sclerosis participants without history of optic neuritis; MS-ON = multiple sclerosis participants with history of optic neuritis; DMT = disease-modifying therapy, with anti-CD20 therapy comprising rituximab (*n* = 4), ocrelizumab (*n* = 2), and ofatumumab (*n* = 1); PDDS = Patient-Determined Disease Steps; VFQ-25 = Visual Function Questionnaire-25; T25FW = Timed 25 Foot Walk; 9HPT = 9-Hole Peg Test; SDMT = Symbol Digit Modalities Test; LCLA = Low Contrast Letter Acuity. HC were matched 2:1 to MS participants on age (± 4 years), with additional matching on sex. * Some participants had very poor visual acuity and were unable to perform these tests. In order to include these eyes, we used a Winsorized estimator, in which a value of 1.1 (equivalent to 20/250 vision) was assigned to eyes that were unable to perform the tests. All Winsorized values were among ON eyes

Disease-modifying therapy at enrollment is summarized in Tables 1 ; 14 of 15 participants (93.3%) were receiving treatment.

Self-reported baseline disability measures showed relatively mild impairment with 66.7% having PDDS score of 0–1, indicating no or mild disability, and the remainder distributed across scores 2–4. Clinical disability measures also indicated only minor abnormalities. Mean T25FW was 4.6 (SD 1.0) seconds, which is within normal limits (normal < 6 s). The 9HPT with dominant hand averaged 21.7 (SD 2.1) seconds, also within normal range (normal < 25 s). SDMT scores averaged 55.5 (SD 7.6), indicating preserved cognitive processing speed (age-adjusted normal range: 45–65). For visual outcomes, BCVA and LCLA are reported in logMAR units where higher values indicate worse performance, while VFQ-25 scores reflect visual quality of life where 100 indicates a perfect score and lower scores indicate worse function. Visual function questionnaire scores averaged 84.1 (SD 12.6) in MS-ON and 89.1 (SD 5.7) in MS-NON participants. Visual acuity was well preserved in both groups with median logMAR of 0.00 in both MS-ON (interquartile range [IQR] 0.00, 0.20) and MS-NON eyes (IQR − 0.10, 0.10), equivalent to approximately 20/20 Snellen acuity. LCLA at 5% contrast showed median scores of 0.30 logMAR (IQR 0.20, 0.30) in MS-NON eyes, indicating preserved low-contrast vision, compared to 0.40 logMAR (IQR 0.40, 0.60) in MS-ON eyes, demonstrating worse performance. At 2.5% contrast, MS-NON eyes showed median 0.70 logMAR (IQR 0.60, 0.70) versus MS-ON eyes at 0.80 logMAR (IQR 0.70, 1.10), with greater impairment at lower contrast levels (detailed scores in Table 1).

### Baseline OCT and OCTA Measures

**Fig. 1:**
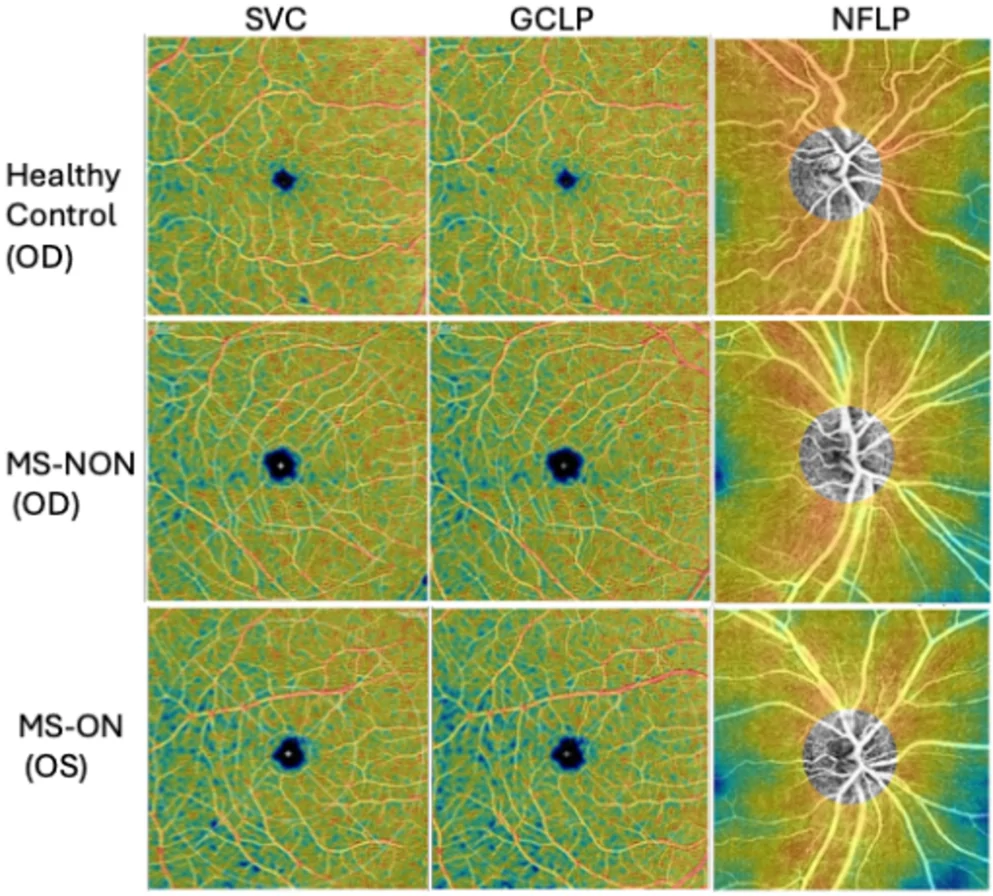
Representative Optical Coherence Tomography Angiography (OCTA) Vessel Density Maps in a Healthy Control and in MS Eyes With and Without Optic Neuritis History. Vessel density maps for macular superficial vascular complex (SVC) and ganglion cell layer plexus (GCLP) and the disc nerve fiber layer plexus (NFLP). Top: healthy control (HC); middle: MS eye without optic neuritis history (MS-NON); bottom: MS eye with optic neuritis history (MS-ON). The MS-NON and MS-ON eyes are from the same participant. The MS-ON map (left eye) is mirrored to right-eye orientation. Warmer colors indicate higher vessel density and blue indicates lower vessel density

**Fig. 2:**
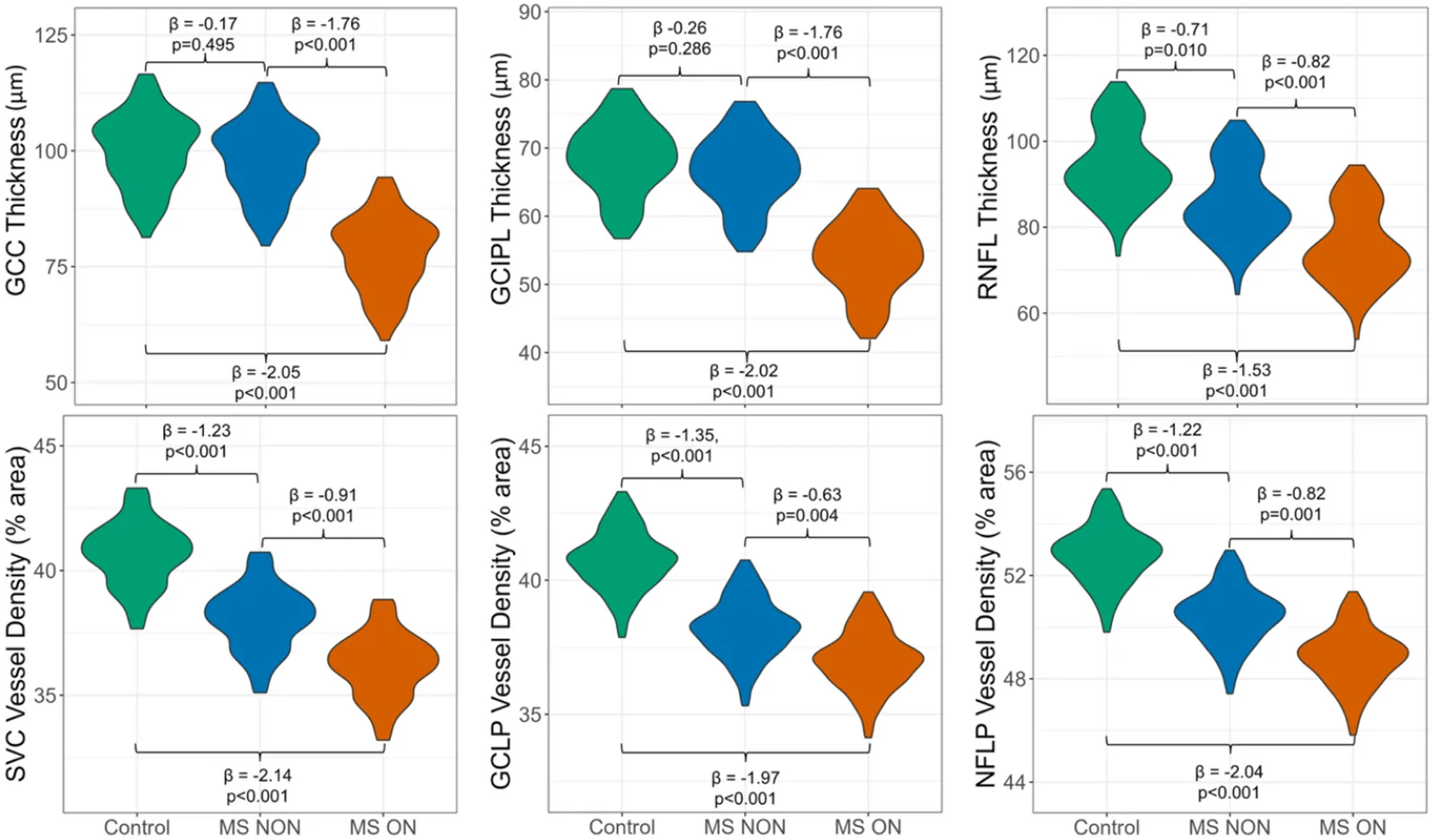
Violin Plots of Optical Coherence Tomography (OCT) and Optical Coherence Tomography Angiography (OCTA) Outcomes in Healthy Controls and Multiple Sclerosis Eyes with and without Optic Neuritis. Structural thickness measures (GCC, GCIPL, RNFL) are shown in micrometers (µm) and vessel density measures (SVC, GCLP, NFLP) as percent area (% area). Standardized β coefficients and p-values from linear mixed-effects models are shown for each pairwise group comparison. GCC = ganglion cell complex, GCIPL=ganglion cell inner plexiform layer, RNFL=retinal nerve fiber layer, SVC=superficial vascular complex, GCLP=ganglion cell layer plexus, NFLP=nerve fiber layer plexus

Representative vessel density maps for each group are shown in Fig. 1. Baseline structural OCT and OCTA measurements comparing MS eyes stratified by ON history to age- and sex-matched HC are shown in Table 2; Fig. 2. MS-NON eyes demonstrated significantly reduced vessel density compared to HC across all retinal layers: SVC by 1.23 SD (*p* < 0.001), GCLP by 1.35 SD (*p* < 0.001), and NFLP by 1.22 SD (*p* < 0.001). In contrast, macular ganglion cell thickness was relatively preserved, with GCC reduced by 0.17 SD (*p* = 0.495) and GCIPL reduced by only 0.26 SD (*p* = 0.286). Peripapillary RNFL thickness was reduced to a greater extent, at 0.71 SD (*p* = 0.010).

**Table 2:** Baseline Retinal Structural and Vessel Density Measures

a: Baseline OCT/OCTA Values, Mean (SD)						
Measure	HC (*n* = 44 eyes)		MS-NON (*n* = 21 eyes)		MS-ON (*n* = 9 eyes)	
**Structural Thickness (µm)**						
GCC	100.1 (7.0)		96.3 (9.3)		78.8 (9.2)	
GCIPL	68.5 (5.0)		64.9 (5.7)		54.2 (5.6)	
RNFL	95.1 (10.3)		84.2 (7.8)		73.4 (10.8)	
**Vessel Density (% area)**						
SVC VD	40.7 (1.3)		37.6 (1.2)		37.1 (1.5)	
GCLP VD	40.8 (1.2)		38.0 (1.1)		37.6 (1.2)	
NFLP VD	52.9 (1.3)		50.4 (1.1)		49.2 (1.3)	

b: Linear Mixed Effects Model Comparisons						
Measure	Comparison	Percent Difference	Standardized β	SE	95% CI	*p*-value
**GCC**	MS-NON vs. HC	-1.83%	-0.17	0.24	(-0.66, 0.33)	0.495
	MS-ON vs. HC	-22.56%	-2.05	0.29	(-2.63, -1.46)	< 0.001
	MS-ON vs. MS-NON	-21.12%	-1.88	0.23	(-2.34, -1.42)	< 0.001
**GCIPL**	MS-NON vs. HC	-2.81%	-0.26	0.24	(-0.75, 0.23)	0.286
	MS-ON vs. HC	-21.79%	-2.02	0.28	(-2.59, -1.45)	< 0.001
	MS-ON vs. MS-NON	-19.52%	-1.76	0.21	(-2.17, -1.35)	< 0.001
**RNFL**	MS-NON vs. HC	-9.66%	-0.71	0.25	(-1.23, -0.18)	0.01
	MS-ON vs. HC	-20.92%	-1.53	0.3	(-2.15, -0.91)	< 0.001
	MS-ON vs. MS-NON	-12.46%	-0.82	0.24	(-1.30, -0.35)	0.001
**SVC**	MS-NON vs. HC	-6.33%	-1.23	0.23	(-1.70, -0.76)	< 0.001
	MS-ON vs. HC	-11.01%	-2.14	0.27	(-2.69, -1.58)	< 0.001
	MS-ON vs. MS-NON	-4.99%	-0.91	0.22	(-1.33, -0.48)	< 0.001
**GCLP**	MS-NON vs. HC	-6.26%	-1.35	0.22	(-1.79, -0.90)	< 0.001
	MS-ON vs. HC	-9.18%	-1.97	0.26	(-2.51, -1.44)	< 0.001
	MS-ON vs. MS-NON	-3.12%	-0.63	0.22	(-1.05, -0.20)	0.004
**NFLP**	MS-NON vs. HC	-4.50%	-1.22	0.21	(-1.65, -0.79)	< 0.001
	MS-ON vs. HC	-7.52%	-2.04	0.27	(-2.59, -1.50)	< 0.001
	MS-ON vs. MS-NON	-3.17%	-0.82	0.25	(-1.31, -0.34)	0.001

(A) Descriptive statistics showing mean (SD) for structural thickness (µm) and vessel density (% area) in age- and sex-matched healthy controls (HC), MS eyes without optic neuritis (MS-NON), and MS eyes with optic neuritis (MS-ON). (B) Linear mixed effects model results showing the percent difference between adjusted group means relative to the healthy control mean, standardized β coefficients, standard errors, 95% confidence intervals, and p-values. MS-NON eyes showed significant RNFL thinning (*p* = 0.010) with relatively preserved macular ganglion cell thickness (GCIPL, GCC; both *p* > 0.10), alongside a significant reduction in vessel density (all *p* < 0.001). MS-ON eyes showed both structural thinning and reduced vessel density (all *p* < 0.001)

In contrast, MS-ON eyes showed both structural thinning and more severe reductions in vessel density compared to HC. VD reductions were even more pronounced than in MS-NON eyes: SVC by 2.14 SD (*p* < 0.001), GCLP by 1.97 SD (*p* < 0.001), and NFLP by 2.04 SD (*p* < 0.001). All structural measures were also significantly reduced: GCC by 2.05 SD (*p* < 0.001), GCIPL by 2.02 SD (*p* < 0.001), and RNFL by 1.53 SD (*p* < 0.001). Direct comparison between MS-ON and MS-NON eyes confirmed significantly greater structural loss (differences of 0.82–1.88 SD, all *p* ≤ 0.001) and vessel density loss (differences of 0.63–0.91 SD, *p* ≤ 0.004) in ON-affected eyes.

### Structure-Vascular Correlations

**Fig. 3:**
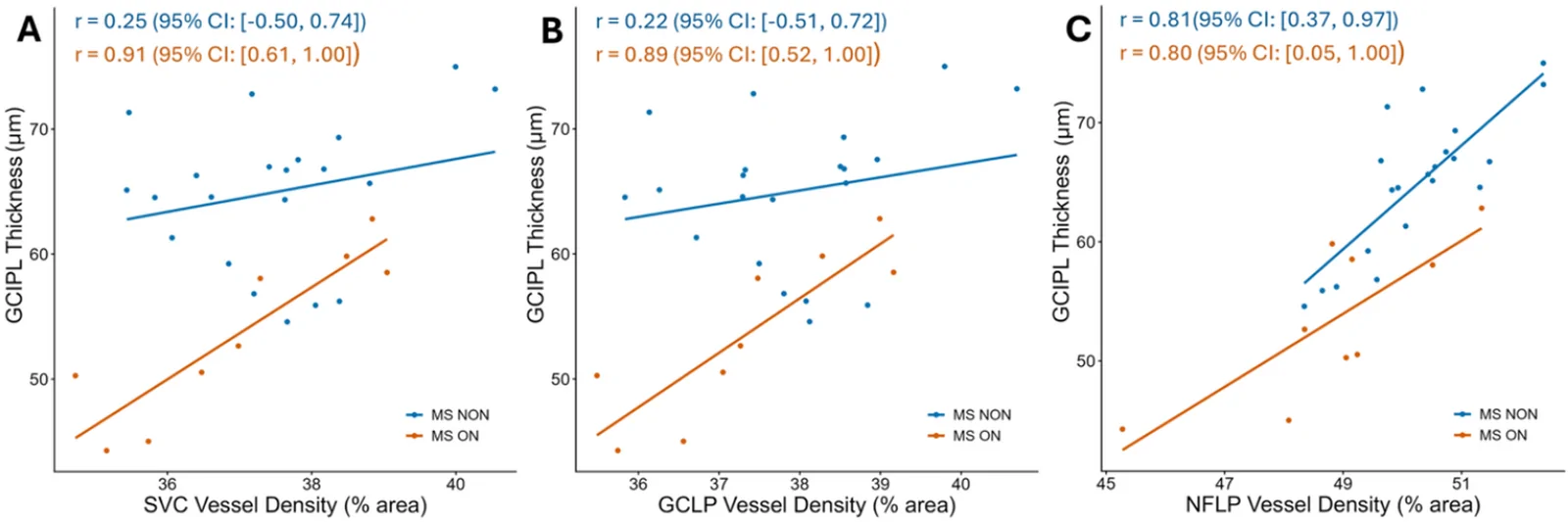
Structure-Vascular Dissociation in MS Eyes With and Without Optic Neuritis History. Scatter plots showing the relationship between ganglion cell–inner plexiform layer (GCIPL) thickness (µm) and retinal vessel density (% area) in MS eyes stratified by optic neuritis history. Pearson correlation coefficients (r) with 95% confidence intervals are shown for MS-NON and MS-ON eyes. GCIPL = ganglion cell–inner plexiform layer; SVC = superficial vascular complex; GCLP = ganglion cell layer plexus; NFLP = nerve fiber layer plexus

We examined correlations between OCTA VD and OCT structural thickness separately in MS-ON and MS-NON eyes (Table 3; Fig. 3). In MS-ON eyes, macular ganglion cell thickness correlated strongly with all three vascular layers (GCIPL with each vascular layer, *r* = 0.80–0.91), consistent with parallel structural and vascular degeneration after optic neuritis.

**Table 3:** Structure-Vascular Correlations

Structural Measure	MS-NON Eyes (*n* = 21)			MS-ON Eyes (*n* = 9)		
	SVC VD	GCLP VD	NFLP VD	SVC VD	GCLP VD	NFLP VD
**GCC**	0.34 (-0.42, 0.79)	0.33 (-0.42, 0.76)	0.81 (0.36, 0.96)	0.88 (0.33, 1.00)	0.85 (0.23, 1.00)	0.75 (0.02, 0.98)
**GCIPL**	0.25 (-0.50, 0.74)	0.22 (-0.51, 0.72)	0.81 (0.37, 0.97)	0.91 (0.61, 1.00)	0.89 (0.52, 1.00)	0.80 (0.05, 1.00)
**RNFL**	0.25 (-0.45, 0.71)	0.17 (-0.55, 0.62)	0.51 (-0.16, 0.92)	0.59 (-0.44, 0.99)	0.59 (-0.46, 1.00)	0.88 (0.19, 0.99)

Pearson correlation coefficients (r) and 95% confidence intervals for correlations between OCT structural thickness measures and OCTA vessel density measures in MS eyes stratified by optic neuritis history. MS-ON eyes demonstrate strong correlations between structural and vessel density measures across all retinal compartments, indicating tightly coupled structural and vascular degeneration following optic neuritis. In contrast, MS-NON eyes show weak correlations for superficial vascular layers (GCLP, SVC), demonstrating structure-vascular dissociation, while NFLP maintains strong correlations with structural measures

In MS-NON eyes, this coupling was absent in the macula. Macular VD (GCLP, SVC) correlated only weakly with ganglion cell thickness (Table 3). NFLP VD was the exception, retaining strong correlations with structural thickness even in these eyes (Table 3), setting it apart from the macular plexuses.

### Correlations with Clinical Outcomes

Correlations between averaged OCT/OCTA measures and clinical outcomes were assessed within the MS cohort (Tables 4 and 5).

#### Visual Function: Eye-Level Visual Outcomes

BCVA correlated weakly with retinal measures in both MS-ON and MS-NON eyes (Table 4). In MS-ON eyes, correlations between visual acuity and retinal measures were generally weak to moderate and non-significant for both structural measures (GCC: ρ =-0.50, 95% CI [-1.00, 0.60]; GCIPL: ρ =-0.59, 95% CI [-1.00, 0.52]; RNFL: ρ =-0.49, 95% CI [-1.00, 0.60]) and vessel density measures (GCLP VD: ρ =-0.35, 95% CI [-1.00, 0.64]; SVC VD: ρ =-0.35,95% CI [-1.00, 0.64]; NFLP VD: ρ =-0.65, 95% CI [-1.00, 0.60]). MS-NON eyes showed weakly positive, non-significant correlations between visual acuity and both structural and vessel density measures (all ρ ≤ 0.34).

**Table 4:** Eye-Level Correlations between Retinal Measures and Clinical Outcomes

	Best-Corrected Visual Acuity (BCVA)	
Measure	MS-NON Eyes (*n*=21)	MS-ON Eyes (*n*=9)
**Structural Thickness**		
GCC	0.34 (-0.27, 0.72)	-0.50 (-1.00, 0.60)
GCIPL	0.32 (-0.27, 0.74)	-0.59 (-1.00, 0.52)
RNFL	0.10 (-0.51, 0.67)	-0.49 (-1.00, 0.60)
**Vessel Density**		
SVC	0.06 (-0.60, 0.62)	-0.35 (-1.00, 0.64)
GCLP	0.21 (-0.39, 0.68)	-0.35 (-1.00, 0.64)
NFLP	0.17 (-0.43, 0.73)	-0.65 (-1.00, 0.29)
	**Low-Contrast Letter Acuity (LCLA 5%)**	
**Measure**	**MS-NON Eyes (** ***n*** **=21)**	**MS-ON Eyes (** ***n*** **=9)**
**Structural Thickness**		
GCC	0.18 (-0.44, 0.67)	-0.94 (-1.00, -0.09)
GCIPL	0.10 (-0.54, 0.66)	-0.82 (-1.00, -0.09)
RNFL	-0.02 (-0.61, 0.58)	-0.82 (-1.00, 0.20)
**Vessel Density**		
SVC	0.47 (-0.23, 0.87)	-0.66 (-1.00, -0.03)
GCLP	0.48 (-0.16, 0.87)	-0.66 (-1.00, -0.03)
NFLP	0.02 (-0.69, 0.62)	-0.59 (-1.00, 0.64)
**Measure**		**MS-ON Eyes (** ***n*** **=9)**
**Structural Thickness**		
GCC	-0.24 (-0.72, 0.37)	-0.94 (-1.00, -0.46)
GCIPL	-0.21 (-0.67, 0.39)	-0.86 (-1.00, 0.00)
RNFL	-0.38 (-0.84, 0.33)	-0.93 (-1.00, -0.45)
**Vessel Density**		
SVC	0.32 (-0.27, 0.77)	-0.63 (-1.00, 0.51)
GCLP	0.21 (-0.40, 0.73)	-0.63 (-1.00, 0.51)
NFLP	-0.34 (-0.75, 0.23)	-0.64 (-1.00, 0.47)
	**Low-Contrast Letter Acuity (LCLA 1.25%)**	
**Measure**	**MS-NON Eyes (** ***n*** **=21)**	**MS-ON Eyes (** ***n*** **=9)**
**Structural Thickness**		
GCC	-0.09 (-0.67, 0.63)	-0.93 (-1.00, -0.46)
GCIPL	-0.05 (-0.66, 0.65)	-0.85 (-1.00, 0.00)
RNFL	-0.24 (-0.74, 0.55)	-0.89 (-1.00, -0.32)
**Vessel Density**		
SVC	0.61 (0.08, 0.92)	-0.62 (-1.00, 0.56)
GCLP	0.57 (-0.05, 0.90)	-0.62 (-1.00, 0.56)
NFLP	-0.17 (-0.77, 0.53)	-0.60 (-1.00, 0.47)

Spearman correlation coefficients (ρ) and 95% confidence intervals for correlations between OCT structural thickness measures, OCTA vessel density measures, and visual acuity outcomes. Visual acuity is reported in logMAR units where higher values indicate worse performance; negative correlations indicate that reduced structural thickness or vessel density is associated with worse visual acuity. MS-ON eyes demonstrate strong correlations between retinal measures and visual function across all contrast levels, with strongest correlations at 2.5% and 1.25% contrast, indicating measurable functional consequences of structural and vascular loss. MS-NON eyes show predominantly weak correlations, particularly for high-contrast (BCVA) and moderate-contrast (LCLA 5%, 2.5%) tests, indicating functional preservation despite reduced vessel density

**Fig. 4:**
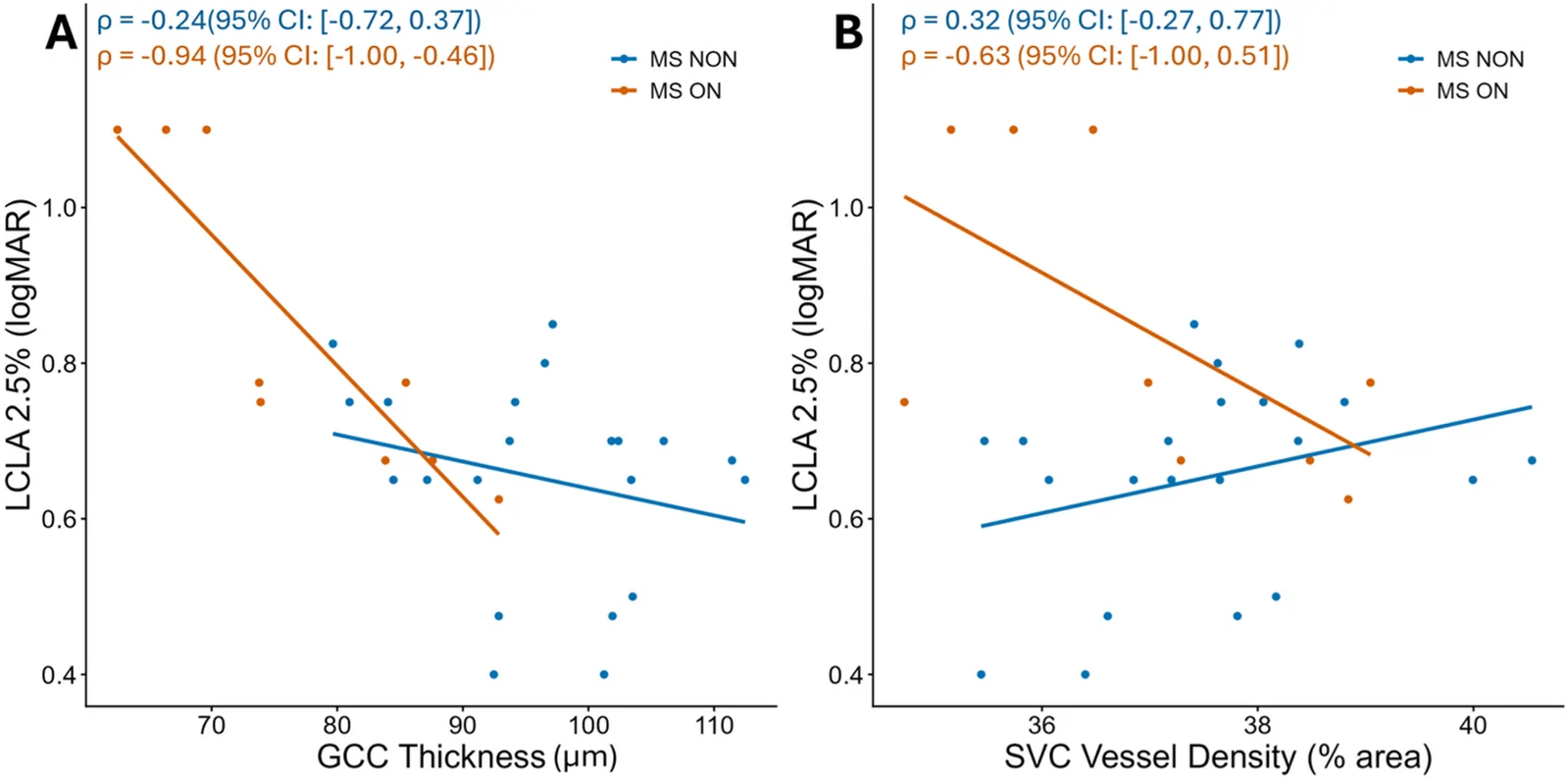
Functional Correlations with Low-Contrast Letter Acuity. Scatter plots showing the relationship between low-contrast letter acuity (LCLA) at 2.5% contrast (logMAR) and retinal measures (GCC thickness in µm; SVC vessel density in % area) in MS eyes stratified by optic neuritis history. Spearman correlation coefficients (ρ) with 95% confidence intervals are shown for MS-NON and MS-ON eyes. LCLA = low-contrast letter acuity; GCC = ganglion cell complex; SVC = superficial vascular complex

In MS-ON eyes, low-contrast acuity correlated with retinal structural thickness at both 5% and 2.5% contrast (Table 4). We focus on 2.5% contrast for its established sensitivity to visual pathway dysfunction in MS, where GCC and RNFL showed the strongest associations (Fig. 4). The remaining correlations were directionally consistent but had wide confidence intervals in this small sample. In MS-NON eyes, where acuity remained near-normal, no measure correlated strongly with vision at any contrast level.

#### Visual Function: Person-Level Visual Outcomes

VFQ-25 composite scores correlated weakly with all retinal measures (Table 5).

**Table 5:** Person-Level Correlations between Retinal Measures and Clinical Outcomes

Outcome Measure	GCC	GCIPL	RNFL	SVC	GCLP	NFLP
VFQ-25ᵃ	0.33 (-0.22, 0.72)	0.33 (-0.22, 0.72)	0.34 (-0.21, 0.73)	-0.31 (-0.71, 0.24)	-0.22 (-0.66, 0.33)	0.41 (-0.12, 0.76)
VFQ Neuro-Ophth Suppl.	0.46 (-0.07, 0.79)	0.54 (0.04, 0.82)	0.50 (-0.02, 0.81)	-0.06 (-0.56, 0.46)	-0.02 (-0.52, 0.50)	0.48 (-0.05, 0.79)
MSFC	0.32 (-0.23, 0.72)	0.25 (-0.30, 0.68)	0.20 (-0.35, 0.64)	-0.13 (-0.60, 0.41)	-0.04 (-0.54, 0.48)	0.14 (-0.40, 0.61)
PDDSᵃ	-0.29 (-0.70, 0.26)	-0.20 (-0.64, 0.35)	-0.17 (-0.63, 0.38)	-0.14 (-0.61, 0.40)	-0.23 (-0.67, 0.32)	-0.46 (-0.79, 0.07)
MS Durationᵃ	0.35 (-0.20, 0.73)	0.42 (-0.12, 0.77)	0.28 (-0.27, 0.70)	0.23 (-0.32, 0.66)	0.03 (-0.49, 0.54)	0.27 (-0.28, 0.69)

Correlations between OCT structural thickness measures, OCTA vessel density measures, and person-level clinical outcomes in MS participants (*n* = 15). OCT and OCTA measures were averaged between both eyes for each participant. Pearson’s correlation coefficient (r) was used for normally distributed continuous variables; Spearman’s rank correlation coefficient (ρ, denoted by ᵃ) was used for non-normally distributed or ordinal variables. Values are reported as the correlation coefficient (95% CI). Generally weak correlations reflect the early disease stage (mean duration 1.1 years) with minimal global disability despite measurable retinal changes. The VFQ Neuro-Ophthalmic Supplement showed stronger correlations with structural thickness compared to vascular measures and the general VFQ-25 composite, consistent with prior work establishing the VFQ Supplement as sensitive for detecting optic nerve dysfunction

The NEI-VFQ Neuro-Ophthalmic Supplement showed the strongest person-level associations. It correlated with structural thickness (GCIPL *r* = 0.54, GCC 0.46, RNFL 0.50) and with NFLP VD (*r* = 0.48), but not with the superficial vascular layers (GCLP *r* = − 0.02, SVC − 0.06). Only the GCIPL association excluded zero in the confidence interval (95% CI [0.04, 0.82]); full results are reported in Table 5.

#### Clinical Disability

Person-level clinical disability measures correlated weakly with all retinal measures, and the MSFC showed no significant associations (Table 5). PDDS and disease duration also showed no significant correlations with any retinal measure (Table 5).

### Longitudinal Follow-up

Longitudinal assessment was limited to MS eyes. Over the 6-month follow-up period, no retinal measure showed significant change in either MS-NON or MS-ON eyes, and there were no significant between-group differences in the rate of change (Supplementary Table S1). RNFL models excluded the eye that developed incident ON during follow-up, because acute inflammatory injury reflects a different process from the chronic change these models were designed to capture. Including that eye moved the MS-ON RNFL estimate from β = −0.26 (*p* = 0.159) to β = −0.41 (*p* = 0.058), the largest longitudinal effect observed for any measure (Supplementary Table S1).

## Discussion

Consistent with our hypothesis, this study of early RRMS demonstrates that retinal structure-vascular relationships depend on optic neuritis history. In MS-NON eyes, VD was reduced in the macular superficial layers (SVC, GCLP) and the peripapillary NFLP, while macular ganglion cell thickness (GCIPL, GCC) was relatively preserved; MS-ON eyes instead showed tightly coupled structural and vascular loss. These VD reductions, though modest in magnitude relative to control means (4.5% to 6.3%, Table 2), produced the most consistent separation from controls (each greater than one SD; −1.23, − 1.35, and − 1.22 SD respectively; all *p* < 0.001), reflecting their low population variance, whereas the macular ganglion cell layers differed little (GCIPL − 0.26, GCC − 0.17 SD; both *p* > 0.05). Peripapillary RNFL also thinned in MS-NON eyes (− 0.71 SD, *p* = 0.010) and, expressed relative to the control mean, was the largest single change we observed (9.7%, Table 2). Peripapillary nerve fiber layer thinning exceeded macular ganglion cell loss, suggesting the axonal compartment is involved early. The basis for this is considered below. This yields two key findings: microvascular reduction can be present in MS-NON eyes without detectable macular neuronal loss, and, in these eyes, a component of that vascular change may precede structural loss.

Several mechanisms could explain this dissociation. First, vascular pathology may be a primary event in MS neurodegeneration. Endothelial dysfunction and blood-brain barrier disruption are established features of MS pathophysiology: histopathological studies show perivascular inflammation in normal-appearing white matter, and longitudinal data from Montorio et al. [33] found reduced retinal vessel density after 2 years while GCC and RNFL thicknesses remained stable. If confirmed, this supports microvascular dysfunction as an early MS disease indicator and potential therapeutic target. Alternatively, the vascular changes may be secondary to neuroaxonal degeneration. Retrograde trans-synaptic degeneration from subclinical posterior visual pathway lesions, common in MS even without optic neuritis, can affect the nerve fiber layer and its perfusion before the macular ganglion cell bodies in MS [34] and after ischemic stroke [35]. Distinguishing a primary microvascular process from vascular loss secondary to degeneration will require longitudinal data testing whether baseline nerve fiber layer and perfusion loss predict later ganglion cell thinning.

The retinal measures that best separated MS-NON eyes from controls correlated only weakly with vision. Macular VD, which showed the most consistent separation from controls, correlated only weakly with patient-reported vision (GCLP *r* = − 0.02, SVC − 0.06). The structural measures and NFLP VD instead had the greatest associations with the NEI-VFQ Neuro-Ophthalmic Supplement (GCIPL *r* = 0.54, RNFL 0.50, NFLP 0.48, GCC 0.46), consistent with prior work establishing the VFQ Supplement as sensitive for detecting optic nerve dysfunction in MS [36]. This pattern fits two partly distinct processes: in MS-NON eyes that see normally, microvascular loss appears early. However, in MS-ON eyes, structural and visual loss tend to co-occur, consistent with retrograde degeneration of the ganglion cell–axonal complex.

This decoupling of macular vessel density from vision is most evident at the person level. Within MS-ON eyes, which showed more variation and more impairment in visual outcomes, structural thickness correlated strongly with low-contrast acuity (GCC and RNFL with 2.5% acuity; Table 4; Fig. 4), while the vascular correlations followed the same direction but with wider confidence intervals in this small subgroup, consistent with prior literature establishing LCLA as a sensitive marker of visual pathway dysfunction in MS [37]. Within MS-NON eyes, where more participants showed normal or near-normal acuity, no measure correlated appreciably with vision. The vascular layers also differed from one another, however. In these eyes, NFLP VD was strongly correlated with structural thickness, whereas GCLP and SVC were not. This pattern fits a nerve fiber layer plexus coupled to the axons it supplies, while the ganglion-cell and superficial plexuses rarefy more independently of the neurons. Whether NFLP rarefaction is secondary to axonal loss or reflects a distinct microvascular process remains unresolved.

These correlation patterns reinforce the conclusion from the group comparisons: in MS-NON eyes, the reduction in vessel density is present without a measurable functional correlate. That these same eyes show flat vision associations reflects, in part, their near-normal acuity: where function barely varies, even a real structure–vision relationship would be hard to detect. With only 15 patients and 30 eyes, these correlations are hypothesis-generating. However, if reduced vessel density is present in eyes that still see normally and retain near-normal macular ganglion cell structure, it could serve as a clinically meaningful early marker of injury.

Our results confirm and extend previous OCTA investigations in MS showing reduced retinal VD in MS, with greater reductions in ON-affected eyes. Two recent meta-analyses documented reduced retinal VD in MS. Mohammadi et al. analyzed 18 studies and confirmed decreased vessel densities in superficial and peripapillary networks independent of ON history [11]. Wang et al. analyzed 13 studies and similarly found significant reductions in vessel density, though these correlated with structural atrophy, raising the question of whether vascular changes are primary or secondary to neuronal loss [12]. In contrast, studies of acute ON recovery show RNFL thinning within three months while vascular dropout was not detected until one year post-ON [38]. Longitudinal studies in people with established RRMS without ON show progressive thinning and reduced vessel density over 3 years, which may indicate vascular dropout secondary to reduced metabolic demand from neuronal loss [39]. However, few longitudinal studies have compared eyes with and without ON history within the same cohort to determine whether the vessel-structural relationship differs based on prior acute demyelination. Our findings of relatively preserved macular ganglion cell structure with reduced VD in MS-NON eyes align with the temporal sequence observed by Montorio et al. in early disease, though definitive confirmation requires a larger cohort with more extended longitudinal follow-up.

If confirmed in larger longitudinal studies, retinal VD measurements could complement structural OCT in early MS monitoring. OCTA is non-invasive, rapid, increasingly available, and less expensive than MRI perfusion techniques. Open questions requiring longitudinal investigation include whether early vessel rarefaction predicts subsequent structural decline or clinical progression, whether VD changes in response to disease-modifying therapies, and whether the structure-vascular relationship differs based on disease duration, ON history, or inflammatory activity.

OCTA vessel density measures the proportion of pixels with decorrelation signal above a detection threshold, so it registers the loss of detectably perfused vessels rather than graded reductions in flow; reduced velocity within patent capillaries would not be captured. Techniques that assess flow directly may therefore detect change earlier. In a murine optic neuritis model, laser speckle flowgraphy detected reduced retinal blood flow at 7 days after immunization, whereas OCTA vessel density reductions in the deeper capillary plexuses were not apparent until 14 days [40]. In people with MS, retinal blood flow velocities measured with a retinal function imager are reduced in eyes both with and without ON history [41]; in an earlier cohort without ON history, these velocities were not related to RNFL thickness [42]. Retinal tissue perfusion is likewise reduced in MS [43]. Doppler OCT yields absolute total retinal blood flow with approximately 8.5% within-visit measurement variation [44], and variable interscan time analysis OCTA yields relative flow speed rather than a binary determination of perfusion [45]. These methods carry their own constraints: laser speckle flowgraphy reports relative rather than absolute flow and is weighted toward the optic nerve head. On this view, the reduced vessel density we observed in MS-NON eyes may mark the point at which capillary flow has already fallen below the threshold for detection, with functional change beginning earlier.

Our study has several limitations. While we designed this study as a longitudinal investigation, the 6-month follow-up interval was too short to detect structural or vascular changes in this early-disease cohort. Measurement variability (quantified by repeatability coefficients) exceeded the magnitude of expected biological change over this timeframe, making it impossible to distinguish true progression from technical variation. We therefore focused on cross-sectional comparisons. This cross-sectional approach cannot establish temporal relationships between vessel and structural changes. The dissociation in MS-NON eyes, with preserved macular ganglion cell structure and reduced VD, suggests that vascular changes may precede macular ganglion cell loss, but we need longitudinal studies (≥ 2 years based on prior OCTA studies in MS [33]) with extended follow-up to confirm this sequence.

How long an interval would be required is not yet established. Montorio et al. reported reduced vessel density after 2 years with stable GCC and RNFL [33], whereas in two RRMS cohorts, Chen et al. found change confined to the deep vascular plexus over approximately 2 years in 60 eyes of 30 patients [46], and no detectable change in any OCTA vessel density measure over 1 year in 88 eyes of 44 patients [47]. Both are group-level comparisons in cohorts larger than ours; because a repeatability coefficient governs change within a single eye, the interval required to monitor an individual eye would be longer still. Interpretation is further complicated by reports from those cohorts that retinal blood flow and tissue perfusion increased rather than declined over follow-up, so change in vascular measures may not follow a monotonic course. The largest longitudinal effect in our own cohort came from the eye that sustained incident optic neuritis (Supplementary Table S1), consistent with these measures registering acute injury over short intervals more readily than chronic change. Establishing the interval at which vessel density becomes a reliable marker of progression will require dedicated longitudinal study.

Our correlation analyses reflect cross-sectional relationships between measures averaged across all study visits, not within-subject longitudinal changes. While averaging improved precision by reducing inter-visit variability, this approach does not assess how individual eyes change over time.

Our sample size (*n* = 15 MS participants) limits statistical power for subgroup analyses and may not capture the full heterogeneity of early MS. The early disease stage (mean duration 1.1 years from symptom onset) likely explains why retinal measures correlated weakly with person-level disability outcomes. At this stage, patients had minimal global disability despite measurable retinal changes. The restricted disease duration also limits our ability to assess whether structure-vascular dissociation persists throughout the disease course or represents a transient early-stage phenomenon.

All measures in this study were whole-region averages, and the sample was too small for reliable comparisons between sectors, so we cannot determine whether some retinal regions are more vulnerable to vessel density loss than others. In a separate cohort from our group, peripapillary nerve fiber layer thinning in MS was greatest in the temporal-upper and temporal-lower sectors, in eyes both with and without prior ON [48]. That study examined structural thickness only. Whether vessel density loss follows a similar regional pattern is a question for larger studies.

Prior optic neuritis was determined primarily by patient-reported history, supplemented by medical record review where records were available, and no confirmatory imaging or electrophysiology was available. Misclassification is therefore possible, and unrecognized prior ON among MS-NON eyes would be expected to exaggerate the differences we attribute to non-ON disease. The structural findings argue against substantial misclassification: MS-NON eyes showed preserved macular ganglion cell thickness, whereas eyes classified as ON showed marked thinning consistent with prior demyelinating injury. Because recent ON was not an exclusion criterion, three participants were imaged within three months of an episode, when post-ON thinning is still evolving and residual disc edema can partially offset measured nerve fiber layer loss. The peripapillary RNFL reduction in MS-ON eyes (− 1.53 SD) was smaller than the macular GCIPL reduction (− 2.02 SD), a pattern consistent with this effect; the RNFL estimate in ON eyes should therefore be regarded as conservative.

Nearly all participants were receiving disease-modifying therapy at enrollment, and with 15 participants we could not stratify by agent or efficacy tier, so any influence of treatment on retinal vascular or structural measures cannot be assessed here. Cumulative treatment exposure was bounded by a mean disease duration of 1.1 years, which limits but does not exclude a treatment effect.

We matched controls on age and sex, prioritizing age matching (± 4 years) over optimizing sex distributions because of the demographic homogeneity of the MS cohort (86.7% female, 93.3% white) compared to our pool of healthy controls. This reflects the regional MS epidemiology of the Pacific Northwest but limits generalizability. The age-matched control cohort had greater racial diversity (40% Asian, 50% white). Normative studies show race-related differences in retinal vascular measures, particularly for NFLP, which may influence some group comparisons. However, the primary finding of structure-vascular dissociation in MS-NON eyes represents an internal comparison within the MS cohort and is unaffected by this matching limitation.

In conclusion, reduced retinal vessel density offal precedence directly, this cross-sectional dissociation supports the hypothesis that in eyes without optic neuritis, vascular changes may precede or occur independently of macular neuronal loss. If confirmed in larger longitudinal studies, OCTA-derived vascular biomarkers could inform approaches to earlier MS diagnosis and therapeutic monitoring.

## Data Availability

The data that support the findings of this study are available from the corresponding author upon reasonable request.

## Declarations

### Use of Artificial Intelligence

The authors used an AI-based assistant (Anthropic Claude) for language editing, formatting, and manuscript preparation. No content was generated autonomously; the authors reviewed all text and take full responsibility for the content of the manuscript.
